# Design, implementation, and reflections on a two-week virtual visual arts and medicine course for third- and fourth-year medical students

**DOI:** 10.1186/s12909-022-03374-y

**Published:** 2022-04-21

**Authors:** Gavisha R. Waidyaratne, Sangri Kim, Joel D. Howell, John David Ike

**Affiliations:** 1 Internal Medicine Residency Program, The Ohio State University, Columbus, OH USA; 2 Neurology Residency Program, McGaw Medical Center at Northwestern University, Chicago, IL USA; 3 Department of Internal Medicine, University of Michigan Medical School, Ann Arbor, MI USA; 4 Department of History, University of Michigan, Ann Arbor, MI USA; 5 Institute for Healthcare Policy and Innovation, University of Michigan, Ann Arbor, MI USA; 6 Medicine Service, VA Ann Arbor Healthcare System, Ann Arbor, MI USA; 7 National Clinician Scholars Program at the Institute for Healthcare Policy and Innovation, University of Michigan, Ann Arbor, MI USA

**Keywords:** Medical humanities, Visual arts, Undergraduate medical education, Pedagogy, Curriculum evaluation, Virtual learning

## Abstract

**Background:**

Medical humanities courses that incorporate the visual arts traditionally require in-person instruction and visits to museums. The COVID-19 pandemic afforded medical educators a unique opportunity to implement and evaluate virtual visual arts programming.

**Methods:**

A two-week, 7-module visual arts and medicine elective course for third and fourth-year medical students was conducted virtually in the Spring of 2021. The course included traditional didactic components as well as a range of hands-on creative art activities including painting, graphic medicine, photovoice, and Kintsugi (Japanese craft). Digital tools including Canvas, Google Jamboard, and Zoom facilitated student engagement. Student feedback was collected through anonymous post-course surveys.

**Results:**

We successfully conducted a virtual visual arts and medicine elective which integrated hands-on creative art activities. Most students “strongly agreed” that remote instruction was sufficient to meet course objectives. However, all students also “agreed” that in-person instruction may promote more in-depth engagement with the visual arts. The hands-on creative art activities were appreciated by all students.

**Conclusion:**

Visual arts-based medical humanities courses can be delivered virtually and can include hands-on creative art activities such as painting. Future visual arts and medicine courses may benefit from incorporating a range of pedagogical methodologies, digital tools, control groups, and pre−/post-course assessments.

## Introduction

Most United States medical schools offer some form of medical humanities programming intended to improve observation skill, promote tolerance for ambiguity, increase empathy, mitigate burnout, and promote other professional and inter-personal traits required for a successful medical career [1–5]. Since the field’s inception in the 1960s, it has grown to include a wide range of disciplines, including the visual arts. In recent years, major professional organizations have called for the formal integration and expansion of arts and humanities related programming into scientific disciplines [6, 7].

Traditionally, visual arts programming requires in-person instruction and visits to cultural institutions such as museums [1, 8]. But in 2020, the COVID-19 pandemic forced U.S. medical schools to transition to virtual (remote) learning [9, 10]. This dramatic shift resulted in significant changes to existing medical humanities programming, especially those utilizing the visual arts. While some visual arts programs *transitioned* to virtual formats, few, if any, visual arts curricula were designed de novo to utilize an online format [11, 12].

In this manuscript, we share insights we gained from designing and delivering a virtual two-week visual arts and medicine elective course for third- and fourth-year medical students at a single United States medical school. Of note, this course included not only didactic components, but several hands-on creative art activities. We hope a discussion of the lessons learned and the digital tools utilized to engage learners will inform future educators wishing to use all available methodologies to engage health professional students with the visual arts.

### Course development

“The Visual Arts and Medicine” was an online two-week elective course co-designed by fourth-year medical students (GW and SK) and internal medicine faculty (JDH and JDI) to introduce third- and fourth-year medical students to the intersection of the visual arts and clinical medicine. All course directors have experience with the visual arts and medical humanities. Joel Howell is the founding director of the University of Michigan Medical Arts Program, which regularly invites medical students and resident physicians to engage with the arts to enhance their clinical abilities. Joel Howell and John David Ike are contributing faculty to the University of Michigan Medical School (UMMS) Path of Excellence in the Medical Humanities, a four-year academic track within the medical school curriculum. Gavisha Waidyaratne and Sangri Kim were medical student participants in numerous Medical Arts Program events but were not members of the Path of Excellence in Medical Humanities.

The elective was composed of seven distinct modules: Ambiguity & Observation; Graphic Medicine and Patient Narrative; The Visual Arts and Medical Education; Bias and the Visual Arts; The Visual Art of Pandemics; Depictions of Health and Illness in Visual Art; and Art Therapy for Patients and Providers (Table 1). The course broadly explored several themes including visual literacy, observation skill, narrative skill (e.g., storytelling, capturing patient narratives), communication, tolerance for ambiguity, bias, empathy, wellness, and resilience. Many of the modules included hands-on creative art activities to deepen understanding of key concepts. Individual sessions ranged in length from 2 to 3 h. Three modules were conducted over the course of two, two-hour sessions (Graphic Medicine and Patient Narrative; Bias and the Visual Arts; and The Visual Art of Pandemics). Course module instructors were recruited from the University of Michigan academic and professional community. All course modules were led by instructors with extensive in-person teaching experience with the visual arts and humanities in their respective domains; none of the modules had been delivered previously in a virtual or in-person format.

**Table 1:** “The Visual Arts and Medicine” course modules, digital tools, and art creation activities

Module	Objectives	Digital Tools	Art Activity
(1) **Ambiguity and Observation**	Improve observational and visual literacy skills through visual arts engagement. Engage with ambiguity intrinsic to visual art and discuss its implications.	Zoom Google Jamboard Canvas	–
(2) **Graphic Medicine and Patient Narrative**^a^	Appreciate the role graphic narrative can play in conveying stories of illness. Explore the ways graphic medicine can be used as a form of art therapy for patients diagnosed with physical and mental ailments. Create a graphic medicine storyboard and a healthcare comic to learn about the importance of narrative in medicine.	Zoom (+ breakout rooms) Google Jamboard Canvas	Comic Strip Creation
(3) **The Visual Arts and Medical Education**	Review the role for incorporating the visual arts and medical humanities into medical education. Engage in visual literacy exercises. Discuss some of the ways the arts can be used to complement clinical practice (e.g., tolerance for ambiguity, developing a moral imagination, illuminating bias, etc.).	Zoom Canvas	–
(4) **Bias and the Visual Arts**^a^	Explore how the visual arts depict and make the viewer aware of conscious and unconscious bias. Discuss the implications of bias in clinical medicine. Use photovoice to illuminate bias.	Zoom Google Jamboard Canvas	Photovoice
(5) **The Visual Art of Pandemics**^a^	Appreciate artists who experienced and responded to global pandemics using the visual arts. Engage in virtual painting lessons to learn basic painting skills including subject choice, composition, color mixing, and brush techniques. Participate in a virtual art show and discuss the arts role for encouraging reflection and cultivating resiliency.	Zoom (+ breakout rooms) Canvas	Virtual Painting Studio + Art Show
(6) **Depictions of Health and Illness in Visual Art**	Explore the visual representations of illness, health, and disease by artists who grappled with various medical diagnoses. Appreciate how larger sociocultural themes including poverty, scientific advances, and inequality manifest themselves in various visual artworks.	Zoom Canvas	–
(7) **Art Therapy for Patients and Providers**	Introduce students to art therapy’s role in building resilience and fostering healing/wellness in patients and providers. Engage in a hands-on art therapy activity.	Zoom (+ breakout rooms) Canvas	Kintsugi + Art Show

^a^Modules conducted over two sessions

The course was available to all third and fourth-year medical students at UMMS and was advertised to eligible students by the authors (GW, SK) and the UMMS curriculum committee. Five fourth-year medical students formally enrolled in the course and two third-year medical students audited the course. Student leads for the course (SK, GW) also attended and participated in each session. Funding for the course was acquired through an Impact Grant, a type of merit-based funding that supports UMMS student projects. The course was sponsored by the University of Michigan Medical Arts Program and the UMMS Path of Excellence in the Medical Humanities. The course was approved by the UMMS curriculum committee.

### Digital tools for remote learning

Several digital tools facilitated student participation and visual arts engagement, including Canvas, Google Jamboard, and Zoom.

#### Canvas – learning management system

Canvas is a Learning Management System that supports web-based and app-based platforms. The use of Canvas for this course was paid for by the University of Michigan. It was used to share the course syllabus, distribute readings, and as a submission portal for course assignments. The discussion board feature enabled students to engage with other students outside of official class time to discuss assignments and course content.

#### Google Jamboard

Google Jamboard is a free web-based collaborative digital whiteboard that can be accessed with a computer, phone, or tablet. Through interactive sessions referred to as “jams,” groups can respond in real-time to posted content, which can include slide shows, images, and documents. Participants in the “jams” are able to edit the whiteboards using a variety of tools including digital sticky notes to post comments and a digital stylus that permits “drawing” on posted content, including artworks, to illustrate points of emphasis or uncertainty. The Jamboard was used during three course modules: Ambiguity and Observation, Graphic Medicine and Patient Narrative, and Bias and the Visual Arts.

#### Zoom

Zoom is a well-known video communication service that includes live-chat, screen-sharing, and breakout rooms. A university-sponsored Zoom subscription was used for all course modules. Microsoft PowerPoint presentations and Google Jamboards were viewable to all participants via Zoom’s screen share feature. Breakout rooms were used to facilitate small-group discussions of various topics and to engage in hands-on creative art activities (painting, graphic medicine, and Kintsugi). Students were asked to keep their video features enabled during each module to promote a seminar-like environment.

### Hands-on virtual creative art activities

Four of the modules – Graphic Medicine and Patient Narrative, Bias and the Visual Arts, The Visual Art of Pandemics, and Art Therapy for Patients and Providers – included hands-on creative art activities.

#### Virtual painting - the visual art of pandemics

**Fig. 1:**
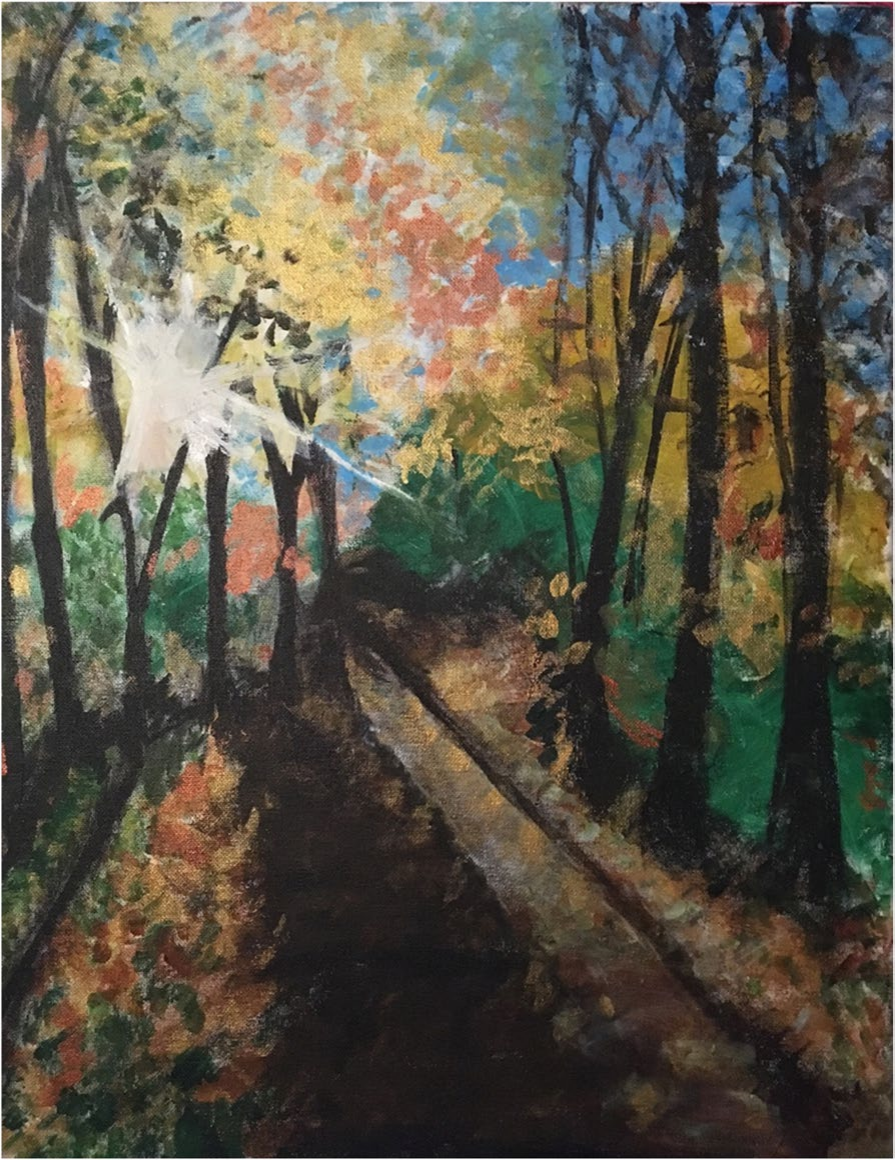
Example painting from the “Visual Arts and Pandemics” module. Reproduced with permission

Following brief didactic lectures on artists who created visual artworks in response to pandemics, including Edvard Munch’s 1918 works *With Spanish Flu* and *After Spanish Flu,* students participated in two virtual painting lessons during “The Visual Art of Pandemics” module. Painting supplies including canvases, brushes, and acrylic paints were delivered to students before the session. Over the course of two, two-hour sessions, the module instructor, a Professor of Medicine who is an experienced painter, guided the students through the basic principles of painting including choosing a subject, organizing a composition, and mixing various paints to achieve a desired color. The Zoom video conference room served as a virtual art studio where students were able to ask their peers or the module instructor specific questions about their artwork. The module concluded with a virtual art show in which students shared their completed paintings (Fig. 1). This also enabled students to discuss how their paintings reflected their own experiences during the COVID-19 pandemic.

#### Healthcare comics – graphic medicine and patient narrative

Over the course of two, two-hour sessions, the “Graphic Medicine and Patient Narrative” module introduced students to graphic medicine as a means to improve communication skill [13]. Students were divided into two small groups and tasked with creating a comic of a meaningful healthcare encounter. During the first session, student groups collaborated in Zoom breakout rooms to draft storyboards, which are organized sequences of distinct scenes and dialogues that contribute to a larger narrative. Students were encouraged to reflect on their clinical experiences when crafting their stories. Module instructors were available to help students select appropriate characters, settings, themes, and narratives to explore in their healthcare-related stories.

During the second session, students collaborated to illustrate the storyboards they drafted during the first session using any two-dimensional creative media (e.g., painting, pencil drawing, crayon, digital media, photograph, etc.). Following the second session, comics were finalized outside of class and submitted on the course’s Canvas page. Students then engaged with one another’s comics through Canvas’ discussion boards to reflect upon the importance of narrative and storytelling in clinical medicine.

#### Photovoice – bias and the visual arts

In session two of the “Bias and the Visual Arts” module, students completed a photovoice assignment. Photovoice is a qualitative research methodology frequently used in community-based participatory research to encourage community reflection and to promote “critical dialogue and knowledge about important issues through large and small group discussions of photographs” [14]. The assignment was designed to help students examine the ways in which the visual arts can unmask underlying biases and how engagement with the visual arts may help improve cultural sensitivity and cultivate empathy. Prior to the session, students took photographs that depicted bias in their daily lives, including people, places, objects, or advertisements. During the session, participants reflected upon the role of conscious (and unconscious) bias in patient care and in their professional development.

#### Kintsugi – art therapy for patients and providers

Following an introductory lecture on the benefits patients and healthcare workers may glean from art therapy, students engaged in a hands-on Kintsugi workshop during the “Art Therapy for Patients and Providers” module. Kintsugi is a Japanese art form that uses lacquers composed of powdered gold, platinum, and other precious materials to repair fractured ceramic pieces. The art form includes philosophical components that explore the beauty that exists within imperfections (*wabi-sab*i) and concepts of non-attachment, acceptance of change, and the fate of human life (*mushin*). Guided by the module instructors, students repaired broken ceramic pieces (e.g., bowls, mugs, plates) that were provided to the students before the session. Each student then shared their completed piece during a virtual art show. This encouraged a discussion about the importance of resilience and overcoming adversity in clinical medicine as well as the beauty that can be found within imperfections.

#### Conducting a virtual hands-on art module

To illustrate the mechanics of conducting a virtual hands-on art activity, we will briefly describe the structure used for each module. Prior to each session, students were emailed a Zoom calendar invitation. The calendar invitation also included specific instructions on how to prepare any necessary hands-on art materials (e.g., rinse and dry new acrylic paint brushes prior to first use, obtain a disposable plate to be used as a paint palate). These instructions were also provided on the course’s Canvas webpage.

During the sessions, module instructors and students positioned their cameras to display their artworks. On rare occasions, students or instructors would utilize two digital devices; one to depict their face and the other to depict their artwork. Both modalities enabled students to ask the module instructors and one another for advice on their works-in-progress. If desired, students also utilized Zoom breakout rooms for one-on-one consultations with module instructors. During dyssynchronous modules, students worked on their artworks outside of official class time. To facilitate sharing during the virtual art shows, students emailed photographs of their completed works to the course directors (GW, SK), which were then presented in a slideshow using Zoom’s screen share feature. This allowed for each student to share reflections on their works of art and promoted group discussion.

### Student feedback

Enrolled students (*n* = 5) provided post-course feedback through an anonymous survey scored on a Likert Scale from 1 (strongly disagree) to 5 (strongly agree). Narrative, open-ended feedback was also collected. Prior experiences with the arts and humanities were also assessed. Students were informed that providing feedback was voluntary, anonymous, and would be included in a research report; all students provided informed consent to these terms prior to the course. The course and its evaluation were approved by the UMMS curriculum committee. All our methods were performed maintaining regulations and guidelines. The response rate to the post-course survey was 100%.

Of those formally enrolled in the course, three students held an undergraduate degree in the arts or humanities and all students reported prior undergraduate courses in the arts and/or humanities (*n* = 5) (Table 2). A single student had participated in the University of Michigan Medical Arts Program and two students were enrolled in the UMMS Path of Excellence in Medical Humanities. All students reported engaging in extracurricular experiences related to the arts and humanities (*n* = 5).

**Table 2:** Prior experiences with the arts and humanities among students enrolled in “The Visual Arts and Medicine” Elective(*n* = 5)

	Proportion of students responding “yes”
Undergraduate major in the arts or humanities	3/5
Undergraduate classes in the arts or humanities	5/5
Past participation in the University of Michigan Medical Arts Program	1/5
Enrollment in the UMMS Path of Excellence in the Medical Humanities	2/5
Other extracurricular experiences with the arts and/or humanities (e.g., arts-related hobbies, play an instrument, visit museums, attend concerts/plays)	5/5

*UMMS* University of Michigan Medical School

Overall, course feedback was positive. On average, most students would recommend the course to their peers (mean score 4.8/5.0) and strongly agreed that the course met their learning expectations (mean score 5.0/5.0) (Table 3). While nearly all students strongly agreed the course was amenable to a virtual format (mean score 4.8/5), they also agreed that the course may have been more impactful if it were held in-person (mean score 4.0/5.0). Students’ narrative feedback disclosed both positive and negative aspects of the elective course (Table 4). Students enjoyed the flexibility of remote learning, but also commented that the course may have been more impactful if it included visits to local art institutions. Students also commented that the course encouraged reflection, listening, and critical thinking. The hands-on art activities were well received.

**Table 3:** Student post-course assessment survey responses for “The Visual Arts and Medicine” elective (*n* = 5)

	Student Responses					
Assessment Questions	1 - Strongly disagree	2 - Somewhat disagree	3 - Neither agree nor disagree	4 - Somewhat agree	5- Strongly agree	Mean (SD)
I would recommend this course to my peers	–	–	–	1	4	4.8 (0.4)
This course met my learning expectations in terms of activity-based and experience-based learning	–	–	–	–	5	5.0 (0.0)
This course expanded my views of the medical arts and humanities	–	–	–	–	5	5.0 (0.0)
This course has made me more interested in pursuing future opportunities in the medical humanities	–	–	–	–	5	5.0 (0.0)
This course should be offered to medical students in the future	–	–	–	–	5	5.0 (0.0)
This course was amenable to a virtual format	–	–	–	1	4	4.8 (0.4)
This course may have been more impactful if it had been held in person	–	–	2	1	2	4.0 (1.0)

**Table 4:** Selected student post-course survey narrative feedback for “The Visual Arts and Medicine” elective (*n* = 5)

Open-Ended Questions	Student Responses
What were the most meaningful or interesting aspects of the course? Why?	“Discussing art, making art, reflecting on experiences throughout medical school -- specifically during the pandemic.” “I loved getting to view different art pieces and reflect on them through discussion and exercises because it helped me to reflect more critically and hear the perspectives of my classmates.” “The hands-on activities (painting, kintsugi, etc.)”
What were your least favorite aspects of this course? Why?	“I wish some aspects could have been in person -- like going to the University of Michigan Museum of Art and the art therapy sessions” “Lecture portions, but they were still good!”
What changes would you make to this course?	“Hopefully segments can be in person in the future. It would be awesome if there were field trips to the University of Michigan Museum of Art and the Detroit Institute of Arts” “Hopefully some aspects can be in person in the future.” “Maybe get rid of one or two sessions that did not have a hands-on component.” “Maybe work on more physical [art] pieces.”
What surprised you about this course?	“How much I loved discussing artwork and paintings.” “[I] learned a lot about the ways art and medicine interact!”
What did you like or dislike about attending the course virtually?	“I like that we could be in our own spaces and comfort of our own homes. However, I wished we could go to the art museum together.” “I thought it was perfect in the virtual format! The only negative was not being able to get more real time feedback on our paintings.” “I liked the flexibility of being able to participate from anywhere, but I miss the opportunity to engage with art pieces in person.”

### Course director reflections

Virtual learning was rarely employed prior to the COVID-19 pandemic. When the pandemic forced a rapid pivot from in-person instruction to remote learning, a small number of medical humanities courses which incorporated the visual arts *transitioned* to a virtual format [11, 12]. This two-week elective was designed de-novo to be delivered virtually.

We were especially pleased that we were able to incorporate hands-on creative art activities into a virtual visual arts and medicine course. These activities included painting, comic strip creation, photovoice, and Kintsugi. Ubiquitous digital tools such as Zoom along with lesser-known technologies including Google Jamboard facilitated direct student engagement with the visual arts. While the virtual creative art activities did not allow the same sort of shared tactile experience that in-person instruction would have offered, it did provide a modicum of richness to what can be a very flat experience over a digital screen. Moreover, students reported in their narrative feedback that the course promoted reflection, critical thinking, and listening to alternate perspectives; these domains have been linked to improved clinical performance, a reduction in burnout, and professional resilience [4, 6, 15].

There were several strengths to delivering the course virtually. First, the virtual format simplified instructor recruitment because it eliminated the need for travel and decreased the time burden on instructors to prepare and present course material. Future virtual (or hybrid) courses could also benefit from recruiting instructors and experts from museums and cultural institutions both locally and elsewhere. Second, similar to the results of a virtual visual arts program at John Hopkins University, our students commented on the ease of learning from the familiar setting of home and a perceived link between remote learning and creativity [12]. In future courses, we may suggest a more expanded series of lectures, assignments, and readings that allows students to further immerse themselves in the visual arts at home prior to in-person engagement with other course participants. Third, we were able to pilot emerging technologies to engage the visual arts. Particularly useful was Google Jamboard, which permitted students to digitally “draw” on works of art, a type of engagement that is impossible within a museum. Future use of these digital tools to complement traditional in-person visual arts instruction may prove useful to medical educators interested in humanities programming, but whenever possible such decisions should be made in conjunction with museum educators and humanities professionals.

While virtual learning has many advantages, it also brings up inevitable challenges. PowerPoint-based virtual lectures can promote “zoning out” and discourage student participation compared to their in-person counterparts [10]. This was reflected in our post-course evaluations, in which students expressed that the course may have been more engaging if it included in-person museum trips or in-studio painting sessions. Although not reported in our course feedback, the virtual interface may have made it more difficult for some students to engage in organic and often vulnerable discussions about art. Moreover, viewing and analyzing visual art with a digital device may have also posed challenges to students whose technological resources were insufficient for appropriate viewing. Overall, a combination of virtual and in-person instruction may provide the flexibility to engage learners who benefit from a variety of instructional methodologies.

### Limitations

There were several limitations to the design, implementation, and evaluation of this virtual visual arts and medicine course. First, the breadth of visual arts and medical humanities programming is much broader than what was covered in our course. Second, we limited our faculty recruitment to those instructors within the local university community. Third, the small number of enrolled students limits the generalizability of student-based course perceptions and feedback. Fourth, two of the digital tools utilized for this course (Zoom, Canvas) require a paid subscription. This cost may limit their use by less-resourced institutions. Fifth, because the class was relatively small, we could not evaluate the use of digital tools and virtual hands-on art activities for larger class sizes. Were the number of students to increase considerably, course directors would need to recruit of additional instructors. Finally, this course lacked evaluation that extended beyond general course feedback. In congruence with the Association of American Medical College’s Fundamental Role of the Humanities in Medical Education initiative and the National Academy of Sciences, Engineering, and Medicine’s aims to craft new pedagogical methods which are evaluated with validated metrics, future iterations of this course could consider including structured qualitative interviews, control groups, and psychometric evaluation [1, 2, 5–7, 16–19].

## Conclusion

Our course demonstrates that virtual visual arts and medicine courses can be successfully designed, administered, and evaluated, and that a virtual course can include hands-on creative art activities. The authors hope the digital tools and experiences presented in this manuscript can serve as a guide to those educators interested in conducting prospective studies of virtual and hybrid medical humanities and visual arts curricula, which could incorporate control groups, pre−/post-course psychometric measurement, and/or inter-institution collaboration. Exploring novel pedagogical approaches that utilize all available virtual and traditional educational tools, when coupled with more formal evaluation, can help enhance teaching of medical humanities.

## Data Availability

All data generated or analyzed during this study are not publicly available due to the materials being student post-course response surveys. The de-identified data is available from the corresponding author upon request. Any potential demographic information (name, date, age, race) will be removed from educational data to ensure compliance with the Family Educational Rights and Privacy Act (U.S. Code Title 20).
